# Emerging Treatment Options for Neuroendocrine Neoplasms of Unknown Primary Origin: Current Evidence and Future Perspectives

**DOI:** 10.3390/cancers16112025

**Published:** 2024-05-27

**Authors:** Francesca Corti, Roberta Elisa Rossi, Pietro Cafaro, Gaia Passarella, Antonella Turla, Sara Pusceddu, Jorgelina Coppa, Simone Oldani, Alessandro Guidi, Raffaella Longarini, Diego Luigi Cortinovis

**Affiliations:** 1 Medical Oncology Unit, Fondazione IRCCS San Gerardo dei Tintori, Via G.B. Pergolesi 33, 20900 Monza, Italy; p.cafaro@unibs.it (P.C.); g.passarella@unibs.it (G.P.); a.turla@unibs.it (A.T.); alessandro.guidi@irccs-sangerardo.it (A.G.); raffaella.longarini@irccs-sangerardo.it (R.L.); diegoluigi.cortinovis@irccs-sangerardo.it (D.L.C.); 2 Gastroenterology and Endoscopy Unit, IRCCS Humanitas Research Hospital, Via Manzoni 56, Rozzano, 20089 Milan, Italy; robertaelisa.rossi@gmail.com; 3 Gastro-Entero-Pancreatic and Neuroendocrine Unit 1, Department of Medical Oncology, ENETS Center of Excellence, Fondazione IRCCS Istituto Nazionale dei Tumori, Via Venezian 1, 20133 Milan, Italy; sara.pusceddu@istitutotumori.mi.it (S.P.); simone.oldani@istitutotumori.mi.it (S.O.); 4 Hepatology and Hepato-Pancreatic-Biliary Surgery and Liver Transplantation Unit, Fondazione IRCCS, Istituto Nazionale Tumori, Via Venezian 1, 20133 Milan, Italy; jorgelina.coppa@istitutotumori.mi.it

**Keywords:** neuroendocrine neoplasms, unknown primary origin, treatment, molecular biology, targeted therapy

## Abstract

**Simple Summary:**

Sufferers of neuroendocrine neoplasms (NENs) of unknown primary origin are a poor prognostic group with largely unmet clinical needs. In the absence of standard therapeutic algorithms, treatment should be based on tumor clinical-pathological characteristics, disease burden, and patient conditions. The aim of this review is to explore the evidence relating to available treatment options for NENs of unknown primary and to offer insights into future perspectives. Particular attention is given to molecular characterization and genomic profiling of NENs with potential therapeutic implications, mainly through the identification of druggable targets for agnostic treatments. Moreover, a treatment algorithm for both well-differentiated and poorly differentiated NENs of unknown primary is proposed.

**Abstract:**

Among neuroendocrine neoplasms (NENs), a non-negligible proportion (9–22%) is represented by sufferers of NENs of unknown primary origin (UPO), a poor prognostic group with largely unmet clinical needs. In the absence of standard therapeutic algorithms, current guidelines suggest that the treatment of UPO-NENs should be based on tumor clinical-pathological characteristics, disease burden, and patient conditions. Chemotherapy represents the backbone for the treatment of high-grade poorly differentiated UPO-NENs, usually providing deep but short-lasting responses. Conversely, the spectrum of available systemic therapy options for well-differentiated UPO-NENs may range from somatostatin analogs in indolent low-grade tumors, to peptide receptor radioligand therapy, tyrosine kinase inhibitors (TKIs), or chemotherapy for more aggressive tumors or in case of high disease burden. In recent years, molecular profiling has provided deep insights into the molecular landscape of UPO-NENs, with both diagnostic and therapeutic implications. Although preliminary, interesting activity data have been provided about upfront chemoimmunotherapy, the use of immune checkpoint inhibitors (ICIs), and the combination of ICIs plus TKIs in this setting. Here, we review the literature from the last 30 years to examine the available evidence about the treatment of UPO-NENs, with a particular focus on future perspectives, including the expanding scenario of targeted agents in this setting.

## 1. Introduction

Neuroendocrine neoplasms (NENs) are a heterogeneous group of rare malignant neoplasms that arise from diffuse neuroendocrine cells. According to the 2022 World Health Organization (WHO) classification, NENs are classified into well-differentiated neuroendocrine tumors (NETs) and poorly differentiated neuroendocrine carcinomas (NECs) based on morphological features and the proliferation rate. Gastroenteropancreatic (GEP) NENs account for 62–67% of cases and include well-differentiated grade (G) 1–2 NETs (Ki-67 ≤ 20%), G3 NETs (Ki-67 index > 20% and well-differentiated morphology), and G3 NECs (Ki-67 index > 20% and poorly differentiated morphology). Thoracic NENs account for 22–27% of cases, including well-differentiated typical carcinoids of the lung and thymus (<2 mitoses/2 mm^2^ and absence of necrosis), atypical carcinoids (2–10 mitoses/2 mm^2^ and/or presence of necrosis), and poorly differentiated small cell (SC) and large cell neuroendocrine carcinomas (LCNECs) (>10 mitoses/2 mm^2^ and presence of necrosis). In the absence of a clinically or radiologically identifiable primary site despite the use of gold standard diagnostic techniques so far, a non-negligible proportion of histologically documented NENs (9–22%) are of unknown primary origin (UPO) [1,2,3,4,5,6].

Compared to other NENs, UPO-NENs represent a significant diagnostic and therapeutic challenge due to their rarity, their complex clinical presentation, and the lack of definite therapeutic algorithms. 

According to the Surveillance, Epidemiology, and End Results (SEER) program, the incidence of UPO NETs was 0.84/100,000 persons per year between 2000 and 2012, with a relatively scant prognosis (median overall survival of 33–48 months) compared to other NET groups [7,8].

UPO-NENs present, by definition, with advanced or metastatic disease, the most frequently involved sites being the liver, followed by the peritoneum, the lymph nodes, the bones, and the lung [9]. Although the gastrointestinal and thoracic origin are the most likely sites of origin of UPO-NENs, unusual locations such as the genitourinary tract or the head and neck district have to be considered [10,11,12,13,14,15,16,17,18,19,20]. 

If the primary tumor site remains unknown despite extensive workup, the initial treatment strategy should be based on the presumptive site of origin and on tumor clinical-pathological characteristics as suggested by the main international guidelines [21,22]. However, in the absence of definite treatment algorithms based on high-quality evidence from randomized phase III trials, an optimal therapeutic approach to UPO-NENs still represents an unmet clinical need.

This review aims to address current evidence about the treatment landscape of UPO-NENs, and to provide an insight into future perspectives, with a particular focus on the potential therapeutic implications of molecular characterization and genomic profiling of these neoplasms.

## 2. Materials and Methods

**Figure 1:**
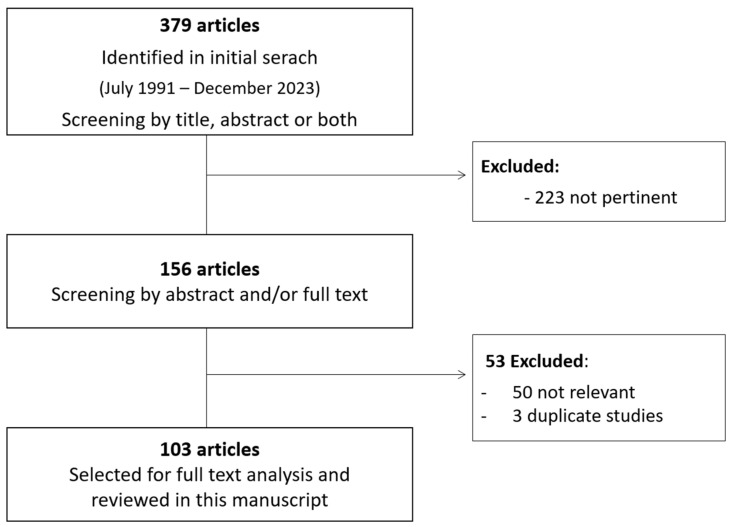
Flow diagram of review manuscript.

We conducted a comprehensive search on PubMed and the ClinicalTrials.gov website using the following search keywords: “neuroendocrine”, with “tumor-” or “neoplasm-”, combined with “unknown primary”, “unknown origin” and “treatment” or “therapy” or “clinical trial” or “molecular biology”. The reference list of the most important papers and abstract communications from relevant conferences were also examined in order to further check the existing data in the literature. We limited the search to English language publications in the last 30 years. Searches were last updated on December 2023. A total of 379 articles were found. A manual selection of relevant articles based on title and/or abstract content was performed. The full versions of all relevant reports were analyzed, with a total of 103 works included in this review. Figure 1 reports the flow diagram for the identification of relevant manuscripts included in the review.

## 3. Diagnostic Approach to UPO-NEN

At the first evidence of UPO-NEN, every effort should be made to identify the primary site of origin, as it could lead to surgical treatment with curative intent and/or to the access to systemic treatment strategies for which primary site identification is required by specific registration boundaries [22]. According to available evidence, the resection of midgut and pancreatic primary tumors is independently associated with improved survival outcomes in NET patients with liver metastases. Moreover, primary tumor surgery may reduce the risk of local complications such as occlusion, bleeding, or perforation, especially in the case of small-bowel NETs [23,24,25,26]. 

A comprehensive assessment for primary site identification should include an extensive clinical evaluation (e.g., pattern of metastatic spread, presence of clinical syndromes), morphological and metabolic imaging, endoscopic procedures, and a thorough immunohistochemical (IHC) evaluation with the possible integration of molecular pathology. Clinical presentation, including the pattern of metastatic organ involvement and the presence of a functional syndrome, might be fundamental as a hint for primary site identification. For example, carcinoid syndrome is usually related to small-bowel NEN, whereas Zollinger–Ellison syndrome and insulinoma and glucagonoma syndromes, should prompt the investigation of the duodenal-pancreatic area, and ectopic ACTH production may suggest lung or thymus primaries [27].

An accurate pathological evaluation is pivotal both to orient primary site identification and to provide tumor grading to guide therapeutic choices. NENs are classified into well differentiated NETs and poorly differentiated NECs based on morphological features and proliferation rate. This classification accounts for critical differences in terms of genomic and biological characteristics, as well as clinical behavior. NETs display a morphological organoid and nesting pattern, with very rare cytological atypia, whereas NECs are characterized by a solid growth pattern with marked atypia and diffuse necrosis. The neuroendocrine phenotype is identified through the assessment of definite immunohistochemical features. NETs usually display Chromogranin A (CgA) and synaptophysin staining, as well as strongly positive somatostatin receptor (SSTR) staining, while NECs retain synaptophysin staining, but may display only focal or absent CgA and SSTR staining. Insulinoma-associated protein-1 (INSM1) is highly specific for NEN independently of primary site and differentiation grade. 

The classification of NENs was recently updated in the 2022 WHO classification system. NECs (every site of origin) are poorly differentiated with a high proliferation rate (Ki-67 > 20%, usually >55%) and further distinguished into the small cell and large cell subtypes. The NET nomenclature has been standardized as a three-tiered grading system according to morphological differentiation, grading, and proliferation rate. GEP NETs are distinguished as well-differentiated low-grade G1 NETs (Ki-67 ≤ 2% and mitotic index < 2 mitoses/2 mm^2^), well-differentiated intermediate-grade G2 NETs (Ki-67 between 3–20% and/or mitotic index between 2 and 20/2 mm^2^), and well-differentiated G3 NETs (Ki67 > 20% and/or mitotic index > 20/2 mm^2^). Similarly, thoracic NETs have been categorized into well-differentiated G1 NETs/typical carcinoids (mitotic index < 2/2 mm^2^ and no necrosis), well-differentiated G2 NETs/atypical carcinoids (mitotic index between 2 and 10/2 mm^2^ and/or necrosis), and well-differentiated NETs with elevated mitotic counts (atypical carcinoid morphology and >10 mitoses/2 mm^2^ and/or Ki67 > 30%). The WHO classification is specifically intended for surgical specimens. Therefore, the limitations of diagnoses obtained from core biopsies or cytological specimens should be taken into account. Whereas the diagnosis of NEC on biopsies may be more reproducible, the diagnosis of NET has inherent limitations in terms of accuracy for the evaluation of the Ki-67 labeling index and mitotic index, potentially affecting tumor grading [1].

Comprehensive IHC evaluation encompassing multiple markers may orient primary site identification. For example, CDX2 is a transcription factor associated with gastrointestinal differentiation, and a possible marker for intestinal or pancreatic origin. CDX2 yields a 90% sensitivity for midgut origin, although it is also expressed in 15% of pancreatic primaries. Paired Box (PAX)-8, PAX6 and Islet-1 are markers for both pancreatic and rectal NENs. Even though Islet-1 is a 70%-sensitive pancreatic (p)NEN marker, it is also expressed in rectal NENs and in up to 10% of lung primaries. Pancreatic and duodenal homeobox (PDX)-1, progesteron receptor (Pr), and neuroendocrine secretory protein (NESP)-55 staining are suggestive of pancreatic primary. Conversely, special AT-rich sequence-binding protein (SATB)2 positivity is typical of rectal (96%) and appendiceal (79%) NETs. Thyroid Transcription Factor (TTF)-1 positivity may suggest a thoracic primary, even though its sensitivity is low, whereas CK7 yields high sensitivity but less specificity for pulmonary NENs. Orthopedia Homeobox Protein (OTP), in contrast, represents a highly specific marker for pulmonary carcinoids, with a 60–80% sensitivity [6,28]. Several IHC algorithms have been built for the presumptive primary site identification in case of UPO-NEN [28,29]. Indeed, sequential IHC staining algorithms may help to identify the presumptive primary site with a stepwise approach. Performing baseline CDX2, Pr, PAX8, TTF-1 and SATB2 staining may lead to the identification of a midgut pattern (CDX2 positive, other markers negative), pancreatic pattern (Pr/PAX8 positive, SATB2 negative, CDX2 positive/negative), rectum/appendix pattern (SATB2 positive, TTF1-negative), or lung pattern (TTF-1 positive) which can be further investigated with the addition of other markers (such as Islet-1, PAX6, OTP, PDX-1, NESP-55) [6,28,29].

Although conventional radiology (computed tomography [CT] or magnetic resonance imaging [MRI]) is of utmost importance for patients’ staging, it might fail to detect the primary tumor if the lesions are small, especially in the case of a GEP primary. Therefore, metabolic imaging represents an essential tool in patient’s staging and primary site detection. Less recent reports evaluated the diagnostic impact of In-pentetreotide somatostatin receptor scintigraphy (SRS) in the detection of GEP occult primaries. In a small series of 36 patients by Savelli and Colleagues, SRS was suggestive of the possible site of the primary lesion in 39% of patients and prompted surgical management in 17% of cases [30]. Due to its higher sensitivity, 68-Gallium [^68^Ga]-labeled somatostatin analogs positron emission tomography (PET) is the gold standard for the detection of low-grade well-differentiated NETs, whereas fluorodeoxyglucose (FDG)-PET is recommended in high-grade poorly differentiated forms and for prognostication. In the case where pheochromocytoma/paraganglioma is suspected, other imaging modalities such as DOPA PET or metaiodobenzylguanidine (MIBG) scanning may also be considered [31,32,33]. According to the available literature, the true positive detection rates for an occult primary with ^68^Ga-DOTA-D Phe1-Tyr3-Octreotide (DOTATOC) PET imaging ranges from 38% to 61%, with overall sensitivity and specificity of 82–92% and 55–82%, respectively. ^68^Ga-DOTATOC PET imaging produces an alteration in patient management in 20–50% of cases [34,35,36,37,38,39]. Moreover, the use of radiolabeled somatostatin analogs has been exploited for intra-operative localization of UPO-NETs suspected to be of GEP primary, to improve intra-operative detection rates of small primaries and/or metastatic sites [40]. 

In cases where morphological or functional imaging has failed to detect the primary site, other investigations should be considered. If a gastrointestinal primary is suspected based on clinical-pathological assessments, endoscopic workout should be performed (including esophagogastroduodenoscopy, colonoscopy, and endoscopic ultrasonography). Since most UPO-NETs are of jejunoileal origin, an accurate study of the small intestine should be performed. Even though data concerning the use of capsule endoscopy (CE) or double balloon enteroscopy (DBE) for UPO-NEN assessment in clinical practice are scant, these assessments may allow the visualization of small intestinal lesions undetectable with conventional radiological imaging. DBE is more invasive when compared with capsule endoscopy; however, it may allow biopsies to be performed to obtain a pathological sample [27]. Moreover, surgical exploration of the abdomen may lead to the identification of the tumor primary in a non-negligible percentage of cases when GEP-NETs are suspected, potentially leading to surgery with radical intent [41].

If other more unusual areas are suspected, a thorough examination of the otolaryngologic and urogenital tracts, as well as full skin, eye, and breast assessments, should be performed [10,11,12,13,14,15,16,17,18,20]. For example, a very aggressive disease with limited therapeutic options is represented by neuroendocrine prostate cancer, which can emerge under the pressure of androgen deprivation treatment or arise de novo in a small percentage of cases [14].

In conclusion, the localization of the primary tumor is of paramount importance for the patient’s management and prognosis. Not only may primary tumor resection have a radical and curative intent, but it has also been associated with improved survival outcomes in midgut and p-NET patients with liver metastases [23,24,25,26]. Moreover, after primary tumor identification and resection, excision or ablation with curative intent of metastatic sites may be pursued as part of a curative strategy (especially in liver-limited disease). Surgery can also decrease the risk of complications related to the primary lesion (such as bleeding, occlusion, or compression of adjacent structures). Finally, identification of the site of origin may allow access to registered systemic treatments which require the primary tumor to be identified [27]. 

**Figure 2:**
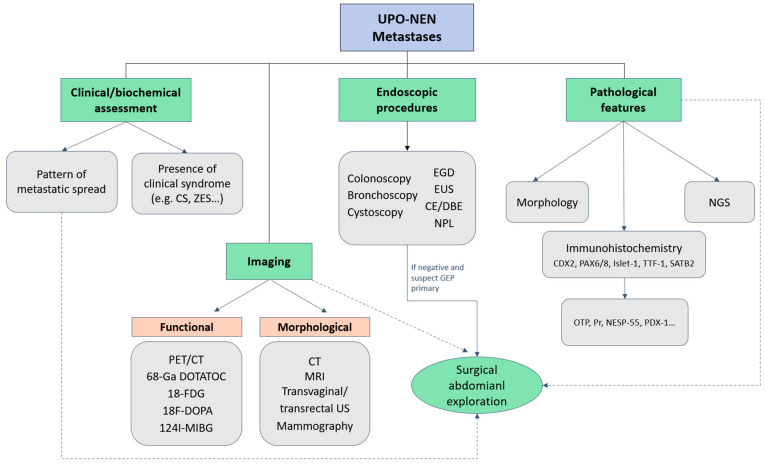
Diagnostic algorithm for UPO-NEN. CE: capsule endoscopy; CS: Carcinoid syndrome; CT: computed tomography; DBE: double-balloon enteroscopy; EGD: Esophagogastroduodenoscopy; EUS: endoscopic ultrasonography; FDG: fluorodeoxyglucose; GEP: gastroenteropancreatic; MIBG: metaiodobenzylguanidine; MRI: magnetic resonance imaging; NESP-55: neuroendocrine secretory protein-55; NGS: next generation sequencing; NPL: naso-pharyngeal-laryngoscopy; OTP: Orthopedia Homeobox Protein; PAX: paired Box; PDX-1: Pancreatic and duodenal homeobox-1; PET: positron emission tomography; Pr: progesterone receptor; SATB2: special AT-rich sequence-binding protein 2; TTF-1: thyroid transcription factor; UPO-NEN: neuroendocrine neoplasm of unknown primary origin; US: ultrasonography; ZES: Zollinger-Ellison syndrome; 68-Ga DOTATOC: ^68^Gallium- DOTA-D Phe1-Tyr3-Octreotide.

Figure 2 depicts a possible diagnostic algorithm for UPO-NEN.

## 4. Therapeutic Approach to UPO-NEN

No definite therapeutic algorithms are currently defined for UPO-NENs, mainly due to the lack of high-quality evidence from randomized phase III trials. 

Therapeutic decision making for UPO-NENs is essentially based on tumor histology and grading, presumptive site of origin (based on histopathological and immunohistochemical characteristics), SSTR status, functionality, tumor burden, and progression rate, as well as the patient’s general condition and comorbidities [21].

### 4.1. Treatment of Poorly Differentiated NEC of Unknown Origin

#### 4.1.1. First-Line Setting

The frontline therapeutic approach to poorly differentiated UPO-NECs is primarily based on the use of platinum-based chemotherapy regimens. In the first-line setting, cisplatin plus etoposide represents the preferred regimen, providing response rates up to ~40–70%, progression-free survival (PFS) rates up to 9 months, and overall survival (OS) rates up to 19 months in historical series [42,43,44,45]. In an analogy with the treatment of SCLC, the combination of platinum agents and irinotecan may be evaluated as a possible alternative to cisplatin and etoposide with some differences in clinical outcomes by ethnicity (Asiatic versus non-Asiatic populations) [46]. A recent study by Zhang and colleagues assessed the non-inferiority of cisplatin plus etoposide vs. cisplatin plus irinotecan in terms of safety and efficacy as a first-line treatment in advanced NEC patients, including UPO-NEC (eight patients), with different toxicity profiles. The overall response rate (ORR) was 42.4% in both arms, with median PFS of 6.4 months and 5.8 months (*p* = 0.81), and median OS of 11.3 months and 10.2 months (*p* = 0.37) for platinum–etoposide and platinum-irinotecan, respectively [47]. The NORDIC-NEC trial retrospectively evaluated the prognostic and predictive factors for survival and treatment outcomes in 305 patients with G3 NEN (32% of whom had UPO-NEN) receiving palliative chemotherapy (including cisplatin-etoposide and carboplatin–etoposide with or without vincristine). ORR to first-line chemotherapy was 31%, with a disease stabilization rate of 33%. The response rates did not differ among different platinum-based regimens. Patients with Ki-67 < 55% had a lower response rate to chemotherapy (15% vs. 42%, *p* < 0.001), but displayed better OS compared to patients with Ki-67 ≥ 55% (14 vs. 10 months, *p* < 0.001) [48]. On the basis of these results, platinum–etoposide containing regimens may not be the most appropriate chemotherapeutic approach for NEN G3 with Ki-67 <55% and alternative regimens (e.g., fluoropyrimidines, temozolomide, and oxaliplatin-containing regimens) should be considered. Recently, the randomized phase II EA2142 trial was designed to assess the efficacy and activity of a regimen with capecitabine and temozolomide (CAPTEM) compared to cisplatin plus etoposide in patients with previously untreated unresectable or metastatic G3 NEN with a Ki-67 labeling index 20–100%. A total of 67 patients with tumors of suspected GEP origin were enrolled. The study was prematurely closed due to futility at 57.7% information time, showing median PFS of 2.43 vs. 5.36 months, OS of 12.6 vs. 13.6 months, and response rate of 9% vs. 10% with CAPTEM and platinum–etoposide, respectively. CAPTEM did not appear to be superior to platinum–etoposide chemotherapy, but was associated with a more favorable toxicity profile [49]. Overall, prospective data assessing G3 NETs independently of G3 NECs are needed to establish the optimal front-line treatment strategy. 

Due to the poor prognosis of these tumors, treatment intensification with three-drug therapeutic regimens has been attempted. The combination of carboplatin, etoposide, and paclitaxel was evaluated in two trials including patients with UPO-NECs, yielding ORR of 47–53% and median OS of 13.4–14.5 months, at the cost of moderate toxicity [50,51]. The FOLFIRINEC randomized phase II trial, with the aim of assessing the efficacy and activity of the FOLFIRINOX (5-fluorouracil, irinotecan, oxaliplatin) regimen compared to platinum plus etoposide in a population of patients with metastatic GEP or UPO NEC, is currently ongoing (NCT04325425) [52].

The combination of frontline chemotherapy with immunotherapy has been recently explored with the aim of improving outcomes in this poor-prognosis population. The non-randomized open-label phase II NICE-NEC trial (EudraCT: 2019-001546-18) evaluated the combination of carboplatin–etoposide with nivolumab as a first-line treatment for patients with advanced or metastatic G3 GEP and UPO NEN. Overall, 38 patients were enrolled. With a median follow-up of 18.6 months (range: 2.2–24.6), ORR was 54.1%, the disease control rate (DCR) was 83.8%, median PFS was 5.7 months (95% confidence interval [CI]: 5.1–9), and median OS was 13.9 months (with 32.4% of patients surviving more than 18 months) [53].

#### 4.1.2. Second-Line Setting

For cases of UPO-NEC progressing on platinum–etoposide, regimens containing irinotecan, fluoropyrimidines, temozolomide, or oxaliplatin (e.g., CAPTEM; FOLFIRI [5-fluorouracil-irinotecan]; FOLFOX [5-fluorouracil-oxaliplatin]) may be considered, mostly based on retrospective data showing ORR rates of ~30% [54,55,56,57]. 

The randomized, multicenter, non-comparative, open-label, phase 2 BEVANEC trial evaluated the efficacy of FOLFIRI plus bevacizumab, or FOLFIRI alone in patients with advanced NEC (including UPO-NEC) progressing on first-line platinum–etoposide-based chemotherapy. Of the 126 patients included in the intent-to-treat population, 18% had UPO-NEC. After a median follow-up of 25.7 months, no clinically significant survival benefit was evidenced with the addition of bevacizumab to the FOLFIRI backbone, with a 6-month OS rate of 53% in the FOLFIRI plus bevacizumab group and 60% in the FOLFIRI group. Moreover, one fatal toxicity (ischemic stroke) occurred in the FOLFIRI-bevacizumab cohort [58]. The NET-02 randomized, non-comparative, phase II trial evaluated the efficacy of liposomal irinotecan (nal-IRI) plus 5-fluorouracil (arm A) or docetaxel (arm B) in patients with poorly differentiated extrapulmonary NECs progressing on first-line platinum–etoposide chemotherapy. Of the 58 enrolled patients, 10% had UPO-NECs. The trial met its primary endpoint in arm A, with a 6-month PFS rate of 29.6% (95% lower confidence limit: 15.7%) that exceeded the prespecified threshold for efficacy, but not in arm B (6 months PFS rate of 13.8%). ORR was 11.1% in arm A and 10.3% in arm B, with similar median PFS (3 months and 2 months) and OS (6 months and 6 months) in patients receiving nal-IRI plus 5-fluorouracil and docetaxel, respectively. According to the authors, 5-fluorouracil plus nal-IRI may represent a viable therapeutic option in this setting, whereas the poor performance of docetaxel, with the poor associated tolerability profile, should discourage further investigation of this regimen in this setting [59]. Another recent phase II trial aimed at evaluating the activity of temozolomide monotherapy in patients with extrapulmonary NECs progressing on first-line platinum–etoposide treatment. The trial enrolled 13 patients, 1 of whom had UPO-NEC. ORR was 15.4%, with median PFS of 1.8 months (95% CI, 1.0–2.7) and median OS of 7.8 months (95% CI, 6.0–9.5). O6-methylguanine-DNA methyltransferase (MGMT) deficiency was observed in one patient, who displayed partial response as the best response [57]. Of note, in the absence of other viable therapeutic options, some authors recently evaluated the opportunity of etoposide rechallenge in patients with a relapse-free interval of ≥3 months after first-line platinum–etoposide treatment. The retrospective RBNEC trial including 121 NEC patients (12% of whom with UPO-NEC) reported DCR of 62%, and median PFS and OS of 3.2 and 11.7 months, respectively, among the 31 patients receiving this treatment strategy [60]. 

Besides standard chemotherapy regimens, other treatment strategies employing immunotherapeutic agents and small molecules have been recently evaluated in order to expand the therapeutic armamentarium in high-grade NENs including UPO-NENs.

As immunotherapy has produced a paradigm shift in the treatment landscape of specific NENs such as Merkel cell carcinoma, immune checkpoint inhibitors (ICIs) have been recently tested in this setting [61,62]. Anti-programmed death (PD)- (ligand [L])1 monotherapy has shown limited activity in molecularly unselected G3 NENs including UPO-NECs, with ORR of 0–7%, median PFS range of 1.8–2.0 months, and median OS range of 4.2–7.8 months [63,64,65,66,67]. The combination of pembrolizumab plus mono-chemotherapy (weekly paclitaxel or weekly irinotecan) in high-grade pretreated extrapulmonary NECs (including 23% of UPO-NECs) also showed unsatisfactory activity (ORR 9%) and poor survival outcomes (median PFS: 2 months, median OS: 4 months) in unselected patients [63,68].

Conversely, dual anti-Cytotoxic T-Lymphocyte Antigen (CTLA)-4/anti-PD-1 blockade yielded more clinically significant results in high-grade NEN patients. The high-grade NEN cohort of the phase II DART-SWOG S1609 trial assessed the activity of the combination of the anti-CTLA-4 agent ipilimumab at the dose of 1 mg/kg every 6 weeks plus anti-PD-1 nivolumab in microsatellite-stable advanced G3 NEN patients (mostly with poorly differentiated tumors). A total of 19 patients were included, 4 (21%) of whom had UPO-NEC. Most of the included patients were pretreated for metastatic disease with a median of 1 (0–3) prior line. ORR was 26% (95% CI, 11–45%), with median PFS of 2.0 months and median OS of 8.7 months. Among the four patients with UPO-NEC, two achieved stable disease as the best response [69]. Another study, the NEN subgroup analysis of the CA209-538 trial of ipilimumab (at the dose of 1 mg/kg every 3 weeks for a total of four doses) and nivolumab in rare cancers, evaluated the outcome of 29 patients with advanced NEN (90% pretreated patients, 45% high grade NEN, 7% UPO-NEN). ORR was 24%, with DCR of 72% in the entire cohort. ORR was 31% and 23% in patients with G3 and G2 NEN, respectively. No responses were observed in the two enrolled patients with UPO-NEC. Median PFS was 4.82 (95% CI 2.71–10.53) months and median OS was 14.78 (95% CI: 4.07–21.25) months [70]. No unexpected toxicities have been documented in both trials combining ipilimumab and nivolumab.

Another ICI combination, durvalumab plus tremelimumab, was evaluated in advanced pretreated NENs in the multicohort phase II DUNE trial, showing only limited activity in advanced G3 GEP and UPO-NENs progressing to platinum-based first-line chemotherapy (cohort 4). In this cohort, ORR was 9.1%, 9-month OS rate was 36.1%, and median OS was 5.9 (95% CI: 2–9.7) months. Even though the prespecified futility threshold for OS was surpassed, response rates and survival outcomes were poor. PD-L1 expression by combined positive score (CPS) did not correlate with treatment activity. In analyzed patients, no microsatellite instability (MSI)-high status was reported [71].

Targeted agents with well-known activity in well-differentiated NENs have also been evaluated in the setting of high-grade progressive NENs. The EVINEC phase II trial evaluated the safety and efficacy of second-line everolimus in NEN G3 patients progressing on platinum chemotherapy. Of the 36 enrolled patients, 13 (36%) had NET G3, 14 (39%) had NEC, and 9 (25%) had mixed-neuroendocrine/non-neuroendocrine neoplasm (MiNEN) with a NEC G3 component. Six patients (16%) had UPO-NEN. No unexpected safety events occurred. Efficacy was promising (ORR 7.7%, median PFS and OS of 5.2 and 23.9 months, respectively) in the NET G3 group. However, results were poor in NECs (ORR 0%, median PFS and OS of 1.8 and 5.6 months, respectively), and in MiNENs (ORR 0%, median PFS and OS of 2.2 and 7.0 months, respectively) [72].

An alternative treatment strategy that is currently being explored in ongoing trials is the combination of small molecules (tyrosine kinase inhibitors-TKIs) and ICIs. Weber and colleagues recently reported the activity and safety of cabozantinib (a c-MET, vascular endothelial growth factor receptor [VEGFR]2, RET, KIT, AXL, and Fms Related Tyrosine Kinase 3 inhibitor) in combination with avelumab in patients with G3 NEN, reporting ORR of 21% and median PFS of 48.1 weeks, with a manageable toxicity profile [73]. Conversely, results of the CABATEN/GETNE-T1914 trial, evaluating the activity of cabozantinib plus the anti-PD-L1 agent atezolizumab, showed limited activity and poor survival outcomes in progressive NENs including high grade NECs [74].

Surufatinib (a VEGFR 1, 2, 3, fibroblast growth factor receptor [FGFR]-1, and colony-stimulating-factor-1 receptor inhibitor), combined with the anti PD-1 sintilimab and the anti-CTLA-4 IBI310, is currently being evaluated for the treatment of high-grade NENs (NCT05165407) [75].

Of note, most of the aforementioned trials included patients with high-grade (G3) NEN, without specifically addressing tumor differentiation. In the future, subgroup analyses assessing NECs independently of G3 NETs are warranted to establish the activity of novel treatment options in these different populations.

### 4.2. Treatment of Well-Differentiated NETs of Unknown Origin

Therapeutic options for well-differentiated UPO-NETs potentially encompass the available agents registered for site-specific disease, ranging from somatostatin analogs (SSAs) to targeted agents (such as everolimus or TKIs), peptide-receptor radio-ligand therapy (PRRT) and chemotherapy. In this setting, therapeutic choices should be tailored according to tumor grading and functionality, SSTR status, tumor burden, and progression rate, as well as the patient’s general condition and comorbidities.

SSA monotherapy may be considered for patients with well-differentiated, low-grade NETs expressing SSTRs, with low tumor burden and/or indolent disease behavior. Moreover, its use must always be considered in functioning tumors for symptomatic control.

UPO-NETs (13%) were included in the pivotal phase III randomized double-blind, placebo-controlled CLARINET trial, evaluating the efficacy of lanreotide in patients with well-differentiated advanced or metastatic, nonfunctioning, SSTR–positive NETs with Ki-67 < 10%. Progression-free survival was significantly improved in the lanreotide group compared to the placebo arm (median PFS not reached [NR] vs. 18.0 months, respectively; hazard ratio [HR] 0.47; 95% CI, 0.30–0.73; *p* < 0.001). Overall survival did not significantly differ between the two arms, although the results’ interpretation may have been impacted by crossover from the placebo to the lanreotide group that occurred in the extension study (CLARINET OLE) and by the long life expectancy of patients with indolent disease [76,77].

Faiss and Colleagues conducted a prospective randomized trial assessing the efficacy of subcutaneous lanreotide (at the dose of 1 mg three times a day), interferon alpha, or their combination in 80 patients with advanced progressive treatment-naïve NETs, including 11 (14%) UPO-NETs, showing a 32% DCR in the lanreotide group [78]. 

More recently, a phase II study in Japanese patients investigated lanreotide autogel in 28 patients with well-differentiated G1–2 NETs including 5 patients (18%) with UPO-NETs, reporting DCR of 64.3%, ORR of 3.6%, and median PFS of 36.3 weeks (95% CI: 24.1–53.1) [79].

PRRT is a valuable therapeutic option for NETs expressing SSTRs, with a good safety profile and limited acute and medium-term toxicities. PRRT employs radiolabeled, beta-emitting SSAs (^90^Yttrium[Y]-DOTATOC and ^177^Lutetium-[DOTA°, Tyr3] octreotate [^177^Lu-Dotatate]) that bind to SSTRs on the surface of tumor cells, with consequent radiopeptide internalization and cell death [80]. The landmark NETTER-1 phase III trial demonstrated the superiority of PRRT with ^177^Lu-Dotatate (in association with octreotide long-acting release [LAR] 30 mg every 28 days) versus high-dose octreotide LAR (60 mg every 28 days) in SSTR-positive midgut NET patients progressing on octreotide treatment, with a significant improvement in terms of PFS and ORR, and a clinically meaningful (~11 months) trend to improved OS in the PRRT arm [81,82]. Even though the phase III NETTER-1 trial only enrolled patients with well-differentiated midgut NETs, the activity of PRRT in other GEP NETs and UPO-NETs has been explored in several retrospective studies. In a meta-analysis of 18 studies considering 1920 patients with unresectable metastatic NETs treated with ^177^Lu-Dotatate, PRRT exhibited a pooled disease response rate of ~30% and a combined DCR of 74–81% [83]. Another systematic review, including one publication addressing eight patients with UPO-NEN, reported ORR of 38% and DCR of 88%, with median PFS of 17.5 months (95% CI 7–34) and median OS of 43 months (95% CI 15 months–NR) [84]. Moreover, data from non-randomized trials of PRRT have consistently shown high response rates and long-term PFS outcomes in heterogeneous patient populations, including UPO-NETs [85,86,87]. PRRT may be the treatment of choice for patients with high STTR expression and/or high tumor burden, in whom tumor shrinkage and symptomatic response, rather than tumor stabilization, represent the therapeutic goal. Moreover, PRRT may also be considered in selected cases in the setting of NEN G3. Data deriving from retrospective studies of PRRT in patients with G3 NEN (including UPO-NEN) highlight disease control rates of 30–80%, median PFS ranging from 9 to 23 months, and median OS ranging from 19 to 53 months. However, reported outcomes were unsatisfactory in patients with Ki-67 > 55% [88,89,90,91]. 

Targeted therapies with demonstrated efficacy in NEN treatment encompass the mammalian target of rapamycin (mTOR) inhibitor everolimus and TKIs. Few data are currently available about the use of targeted therapies in UPO-NENs.

Yao and Colleagues evaluated the activity of everolimus (at the dose of either 5 mg/day or 10 mg/day) in combination with octreotide LAR in 60 patients with well-differentiated NETs, including 5 patients with UPO-NETs (8%). In the intent to-treat population, ORR was 20% (13% in the 5 mg cohort and 30% in the 10 mg cohort). Median PFS was 60 weeks (95% CI, 54–66 weeks), with a 3-year OS rate of 78% [92]. The Italian Trials in Medical Oncology (ITMO) trial enrolled 50 patients with treatment-naïve NETs (including 14 patients with UPO-NETs) to receive octreotide LAR plus everolimus. ORR was 18%, with a complete response rate of 2%, a partial response rate of 16% (including three patients with UPO-NETs), and a disease stabilization rate of 74%. In all patients experiencing a clinical benefit, disease control lasted more than 6 months. In the 5-year updated analysis of this study, 17 (34%) of patients had received treatment for more than 2 years, with a median time to progression of 33.6 months (95% CI 18.7–41.2) and a median OS of 61.0 months (95% CI 49.8-NR) [93].

A subgroup analysis of the RADIANT-4 trial of everolimus vs. placebo in G1–G2 advanced non-functional NETs, specifically focusing on patients with gastrointestinal carcinoids (175 patients) and UPO-NETs (36 patients), showed a clinically meaningful PFS advantage (13.6 months versus 7.5 months) for everolimus vs. placebo (HR 0.60; 95% CI, 0.24–1.51) in the UPO-NET setting [94].

The SANET-ep trial evaluated the efficacy and safety of surufatinib in patients with well-differentiated advanced extra-pancreatic NETs, including 27 (14%) UPO-NETs, evidencing a PFS advantage of surufatinib over placebo (9.2 vs. 3.8 months, HR 0.33; 95% CI, 0.22–0.49, *p* < 0.0001). A specific subgroup analysis of patients with UPO-NETs or NETs of uncommon tumor origin showed median PFS of 13.9 vs. 7.4 months (HR 0.5, 95% CI 0.24–1.06, *p*: 0.069) in the surufatinib and placebo groups, respectively [95].

More recently, the double-blinded phase III Alliance A021602-CABINET trial evaluated the efficacy of cabozantinib versus placebo in patients with advanced NETs progressing on prior therapy. In the extra-pancreatic NET cohort including patients with UPO-NETs, despite modest ORR rates (4% vs. 1% for cabozantinib versus placebo), a statistically significant improvement in PFS (8.2 vs. 3.2 months, HR 0.41, 95% CI 0.27–0.62, *p* < 0.0001) was evidenced in the cabozantinib group over the placebo arm [96].

Cytotoxic chemotherapy also remains an option for patients with well-differentiated UPO-NETs and may be the preferred treatment strategy in patients with high disease burden, higher Ki-67, poor ^68^GaPET, and/or significant FDG-PET uptake, or in patients for whom rapid tumor shrinkage is a desirable goal. In fit patients, polychemotherapy is a preferred option over mono-chemotherapy in terms of activity. Regimens containing alkylating agents (streptozotocin, temozolomide), fluoropyrimidines (5-fluorouracil, capecitabine), oxaliplatin, and irinotecan proved active in NETs. 

Chan and colleagues evaluated the activity of temozolomide in association with bevacizumab in 34 patients with carcinoids, including 7 UPO-NETs (21%), and pNETs. ORR was 15% in the whole study population, even though all responses occurred in the pNET cohort and none in the carcinoid group. However, 12 patients in the carcinoid cohort (63%) experienced some degree of tumor shrinkage. Median PFS was 7.3 months (95% CI, 3.9-NR) and median OS was 18.8 months (95% CI, 8.5–36.1) for carcinoid tumors [97]. Chauhan and colleagues reported median PFS of 10.8 and 7 months, respectively, on CAPTEM in G2 and G3 UPO-NETs [98]. A retrospective real-world experience on 170 patients with GEP, lung, and UPO NETs treated with the CAPTEM combination or temozolomide monotherapy showed clinically meaningful ORR (15.4%) and median PFS (16.9 months, 95% CI 6.0–30.4) and OS (35.7 months, 95% CI 16.2–63.0) results among the 16 (9%) patients with UPO-NETs (4 of whom were diagnosed with NET G3) [99]. Although higher response rates have been reported in patients with MGMT deficiency or promoter methylation treated with temozolomide, the use of MGMT status for preselection of patients remains controversial [99,100,101]. Recently, Walter and colleagues showed increased ORR, PFS, and OS outcomes in patients with methylated compared to unmethylated MGMT NETs (including UPO-NETs) treated with alkylating agents, but not oxaliplatin-based chemotherapy [102].

The NET-01 study randomized 86 patients with advanced GEP and UPO NETs to receive capecitabine and streptozotocin with or without cisplatin. ORR was similar between the two arms (12% vs. 16%), as well as median PFS (10.2 vs. 9.7 months) and OS (26.7 vs. 27.5 months) for the doublet vs. triplet arm, respectively. However, patients enrolled in the triplet group experienced a non-negligible proportion of G ≥ 3 adverse events (68%), compared to the lower rate of high-grade toxicities of patients receiving capecitabine and streptozotocin (44%) [103].

Another valuable treatment option in the setting of well-differentiated NETs is represented by platinum derivates (oxaliplatin) in combination with fluoropyrimidines, with reported ORRs of ~13–30% and median PFS outcomes of 8–20 months [104,105,106].

Immunotherapy has yielded poor results in well-differentiated NENs, with only modest signals of activity in thoracic NENs. Translational studies are needed in order to identify subgroup of patients who are more likely to respond to this treatment strategy [66,70,107,108]. Only the anti-PD-1 agent toripalimab yielded promising response rates (ORR 20%) in Asian patients with G2–3 pretreated NEN. The subgroups of PD-L1-positive, tumor mutational burden (TMB)-high, or *ARID1A*-mutated tumors were enriched in responders [109]. 

**Figure 3:**
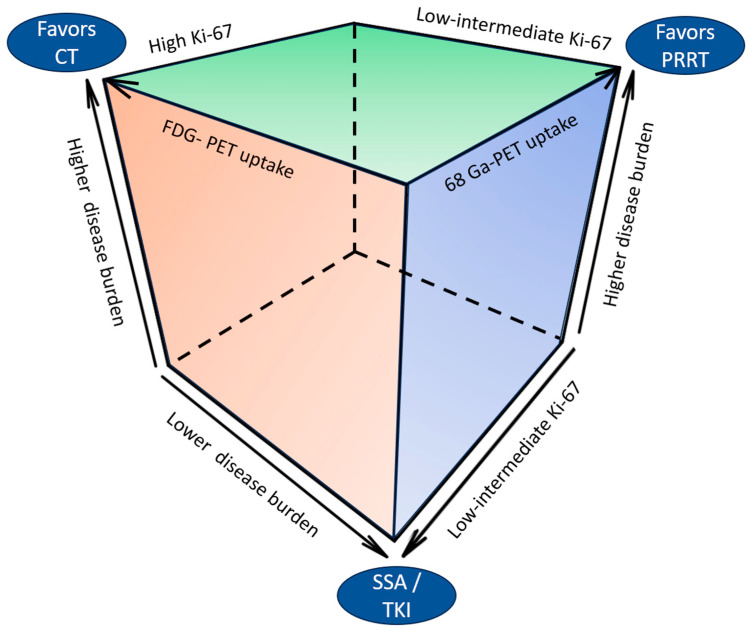
Clinical and pathological determinants of therapeutic choices in UPO-NEN. CT: chemotherapy: FDG: fluorodeoxyglucose; PET: positron emission tomography; PRRT: peptide receptor radioligand therapy; SSA: somatostatin analog; TKI: tyrosine kinase inhibitor.

Figure 3 depicts the clinical and pathological determinants of therapeutic choices in UPO-NEN.

Whenever possible, due to the exiguity of the therapeutic armamentarium and country-specific restrictions in the use of approved treatment options, patients should be considered for enrollment in clinical trials. Table 1 reports ongoing clinical trials including patients with UPO-NEN.

### 4.3. Special Situations: Liver-Limited Disease

Even in the case of UPO-NETs, locoregional treatments may be employed as part of the therapeutic strategy. In particular, liver-directed locoregional therapies, alone or in combination with systemic treatments, may be considered in selected cases to improve local disease control, reduce hepatic tumor burden, and possibly improve patient prognosis [110]. Kim and colleagues conducted a phase 1b dose-finding study of pasireotide, everolimus, and selective internal radioembolization therapy (SIRT) in 13 patients with NET and secondary liver involvement (including 2 patients with UPO-NETs). No dose-limiting toxicities were reported; median PFS was 18.6 months and OS was 46.3 months [111]. Another study of systemic 5-fluorouracil combined with ^90^Y-SIRT in 34 NEN patients with liver metastases showed radiologic ORR of 50% and mean survival of 27.6 months. Among enrolled patients with UPO-NEN, 3 out of 8 (37.5%) experienced a radiologic response [112].

In patients with liver-limited disease, several potentially curative treatment options may be considered, including liver resections or liver transplant. Few cases of liver transplant in patients with UPO-NET have been reported, with alternate outcomes. More data are needed in order to possibly extend the transplant strategy in the setting of UPO-NEN, provided an accurate selection based on patient features, disease behavior, and characteristics [113,114].

## 5. Unraveling Molecular Characterization of UPO-NENs: Time for an Agnostic Approach?

Molecular biology and gene expression profiling represent an area of increasing interest for the characterization of UPO-NENs, with both diagnostic and potential therapeutic implications. In recent years, a deeper insight has been achieved in subtype-specific NEN molecular landscape characterization. These findings, if appropriately integrated with clinical and pathological data, may help to determine tumor origin in UPO-NENs and/or identify druggable molecular targets.

With regard to GEP-NENs, a recent comprehensive genomic profiling analysis confirmed the differences in terms of genomic background of high grade versus low grade GEP-NENs. Among low-grade tumors, the most frequently mutated genes are *ATRX* (13%), *ARID1A* (10%), and *MEN1* (10%), whereas high-grade tumors exhibit *TP53* (51%), *KRAS* (30%), *APC* (27%), and *ARID1A* (23%) mutations and a higher prevalence of *BRAF* (5.4–70%) alterations. Moreover, immune-related biomarkers such as the MSI-high status (4–12% vs. 0–3%), PD-L1 overexpression (6% vs. 1%), and high TMB (7% vs. 1%) are prevalent in high-grade compared to low-grade tumors [115,116]. Among NENs of pancreatic primary, loss of DAXX or ATRX protein expression defines well-differentiated NETs, whereas abnormal p53, Rb, and SMAD4 define poorly differentiated NECs [117]. 

Similarly to GEP-NENs, genomic profiling provides further insight into thoracic NENs biology. Carcinoids generally display low TMB (<1 mutations/megabase [Mb]) and few recurrently mutated genes, including alterations in chromatin remodeling genes and genes in the phosphoinositide 3-kinase (PI3K)/Akt/mTOR pathway. Indeed, mutations in genes involved in chromatin remodeling are detected with a high frequency (~40%) in carcinoids, the most frequent being *MEN1* (11–22%), genes of the SWI/SNF complex mostly affecting *ARID1A* (6–7% of cases) and *KMT2C/MLL3* (8%). Other frequently mutated genes in carcinoids include *TP53* (10%), *NOTCH2* (5%), and *PIK3CA*. Of note, atypical carcinoids usually carry more alterations in the *MEN1* (22% vs. 6%) and *PIK3CA* genes (39% vs. 13%) compared to typical carcinoids. Furthermore, amplification of *MYCL*, *BCL2*, and *SRC* is almost exclusively described in atypical carcinoids [118]. Small cell NECs and LCNECs have higher TMB rates (8.5–10.5 mutations/Mb) compared to carcinoids. The most frequently described LCNEC alterations lie in tumor suppression genes such as *TP53, RB1*, and *STK11*, and genes of the RAS pathway. Indeed, two mutually exclusive genomic subtypes have been identified in LCNECs: the first, which is similar to SCLC, shows concurrent mutation of *TP53* and *RB1*, whereas the other subtype, more similar to non-small cell lung cancer, is predominantly *RB1* wild-type, harboring *STK11/KEAP1* alteration [119]. Preliminary data indicate that *RB1* wild-type tumors may have better outcomes when receiving NSCLC-type chemotherapy (platinum–gemcitabine or paclitaxel) than platinum–etoposide SCLC-like chemotherapy [118]. 

Thus, genomic profiling may provide insights into tumor biological characteristics that segregate with the anatomic primary site and guide therapeutic choices. For example, pulmonary and pancreatic NENs are frequently mutated in chromatin-remodeling genes, whereas midgut NETs exhibit cell cycle and Wnt pathway mutations, such as *CTNNB1*, *MEN1*, or *APC* alterations [29]. Recently, commercially available gene-expression profiling platforms have been implemented with the aim to assist clinicians in the prediction of tumor origin in cases of diagnostic uncertainty [120]. A validated real-time polymerase chain reaction 92-gene cancer ID analysis, able to categorize NENs according to putative primary site and differentiation, was recently shown to yield accuracy in primary site identification of metastatic UPO-NENs, with a likely impact on patient treatment and outcome [120,121]. As previously pointed out, NETs have a lower number of somatic mutations compared to epithelial tumors, while epigenetic modifications due to the mutation or loss of expression of chromatin modifiers are more common. Methylation array data have recently been used in order to create an algorithm for predicting the tissue of origin in UPO-NENs, based on a training set of 97 pNENs and small intestinal NENs [6]. 

Extensive molecular characterization through commercially available next generation sequencing (NGS) platforms may also lead to the identification of druggable targets for approved “agnostic” treatments. Potentially targetable rare molecular alterations in NENs, including UPO-NENs, encompass *BRAF* and *KRAS* mutations, *RET*, Anaplastic Lymphoma Kinase (*ALK*) and Neurotrophic tyrosine receptor kinase (*NTRK*) rearrangements, Delta-like ligand 3 (DLL-3) expression, and MSI-high or TMB-high status. 

*BRAF* V600E mutations, although rare overall in NENs, are usually enriched in high-grade NENs of GEP origin (being reported in up to 70% of right-colon NECs) and have been associated with promising activity of combined BRAF-MEK inhibition [116]. The Food and Drug Administration (FDA) recently approved combined BRAF/MEK inhibition with dabrafenib and trametinib for the treatment of patients with advanced solid tumors harboring *BRAF* V600E mutations, based on the outcomes of basket trials enrolling patients with 24 tumor types (including 2 patients with MiNEN and 2 patients with colon NEC) [116]. Different case series have reported clinically significant responses in pretreated NEC patients harboring *BRAF* V600E mutations with BRAF/MEK targeted agents, even though no specific information about UPO-NENs has been reported so far to our knowledge [122,123,124]. Moreover, in a recent report, a LCNEC patient harboring a non-V600E *BRAF* activating mutation (G469R) showed durable disease control (>15 months) with the combination of dabrafenib and trametinib [125].

Since the development of the KRAS G12C inhibitors sotorasib and adagrasib, few case reports have addressed the potential use of these molecules in the setting of NENs harboring *KRAS* G12C mutations. One case, showing some clinical benefit of sotorasib in a patient with *KRAS* G12C mutant atypical lung carcinoid, has been recently reported [126,127]. More data are required to address the impact of KRAS inhibitors in the setting of NENs, and patients whose tumors harbor *KRAS*-actionable mutations should be referred for enrollment in clinical trials.

*ALK* fusions seem characteristic of thoracic NENs, with a reported incidence of ~0.9–3%, and correlate with high-grade and advanced stages. Few case series have reported significant disease responses of crizotinib and alectinib in lung NECs harboring *ALK* fusion, with a manageable toxicity profile [128,129,130,131]. 

In a large dataset of 2417 NEN patients, the incidence of *NTRK* rearrangements was 0.3% (including 2 patients with UPO-NEN) in the absence of site-specific prevalence [132]. Recently, entrectinib and larotrectinib received agnostic FDA approval for advanced adult or pediatric tumors bearing *NTRK* fusions based on results of the ALKA-372-001, STARTRK-1, and STARTRK-2 trials. ORR for the five patients with NENs enrolled in these trials was 40% [116,133]. Updated results of entrectinib in 121 patients with *NTRK*-rearranged solid tumors showed ORR of 61.2%, median PFS of 13.8 months (95% CI 10.1–19.9), median OS of 33.8 months (95% CI, 23.4–46.4), and intracranial ORR of 63.6% in the whole population. Among the five patients with NEN, ORR was 40%, median PFS was 15.6 months (95% CI 0.9-not estimable), and median OS was 40.5 months (95% CI 28.6–40.5) [134]. In a phase I trial of taletrectinib, a ROS1/NTRK inhibitor, 1 partial response (8.3%) and 7/12 (58%) disease stabilizations were observed among 12 molecularly unselected NEN patients, with median PFS of 10.2 months [135]. 

*RET* alterations include mutations (typical of medullary thyroid cancer and MEN2-related tumors) and rearrangements. The NCT03157128 and NCT03037385 studies of solid tumors (including NENs) harboring *RET* alterations are currently evaluating the safety and activity of the selective inhibitors selpercatinib and pralsetinib, respectively. Pralsetinib is a RET kinase inhibitor, including RET fusion proteins. The phase I/II ARROW trial reported the activity of pralsetinib in patients with *RET*-fusion-positive solid tumors, evidencing 67% ORR among the three enrolled patients with NEN [136]. Other case reports described the clinical activity of selpercatinib and cabozantinib in patients with NECs harboring *RET* alterations, underlying the possibility of an agnostic approach with anti-RET drugs in patients with *RET*-positive NEN [137,138].

DLL3, a negative regulator of Notch signaling, is frequently overexpressed in poorly differentiated NECs, but not in well-differentiated NETs [139]. A DLL3-targeted antibody-drug conjugate linked to a pyrrolobenzodiazepine dimer toxin, Rovalpituzumab tesirine, achieved a 17% ORR in 35 DLL3-overexpressing NEN (NEC and NET) patients (including UPO-NEN) in a phase I/II trial. Median PFS was 4.3 (2.7–6.1) months and median OS was 7.4 (5.6–13.1) months. [140] Further data are, however, required to fully elucidate the effectiveness of Rovalpituzumab tesirine in this setting, as this drug failed to demonstrate an OS benefit over placebo in SCLC patients as maintenance after platinum-based therapy [141] and over topotecan in second-line setting in phase III trials, at the cost of significant toxicity [142]. The DLL3-targeting T Cell engager BI 764532 is currently being evaluated in the DAREON™-5 open-label, multicenter phase II dose-selection trial, in patients with relapsed/refractory neuroendocrine carcinomas (NCT05882058).

With regard to immunotherapeutic agents, few data are currently available about prognostic and/or predictive immune-response biomarkers. In recent years, the anti-PD-1 agent pembrolizumab received agnostic FDA approval for pretreated solid tumors bearing the MSI-H phenotype or a high TMB status (≥10 mutations/Mb). The prevalence of the MSI-H phenotype has been reported in up to 12% of NEN cases, being enriched in cases in gastrointestinal NECs and MiNEN [143].

The updated results of cohort K of the phase II Keynote 158 trial considering 351 patients with MSI-high/deficient mismatch repair (dMMR) non-colorectal cancers, including 12 (3.4%) patients with NEN, showed 30.8% ORR, with a median duration of response of 47.5 months, median PFS of 3.5 (95% CI 2.3–4.2) months, and median OS of 20.1 (95% CI 14.1–27.1) months in the entire cohort, with a manageable safety profile. No separate data on the 12 NEN patients have been specifically reported [144]. These results were recently confirmed in the tumor-agnostic cohort of the DRUP trial (NCT02925234), evaluating the activity of the anti-PD-L1 agent durvalumab in heavily pretreated patients with dMMR/MSI-H solid tumors, including one patient with NEN. ORR was 29%, median PFS was 5 months (95% CI 2-NR), and median OS was 26 months (95% CI 9-NR) in the entire cohort [145]. The Keynote-158 (NCT02628067) phase II study and the NCT04272034 phase I study are currently evaluating the activity and safety of the anti-PD-1 agent pembrolizumab and the anti-PDL-1 inhibitor INCB099318, respectively, in patients with MSI-H solid tumors (potentially including NENs).

High TMB has been detected in up to 45.6% of LCNEC, 11.8% of colon NEN, and 5.9% of patients with small intestinal NEN [146]. Agnostic approval of pembrolizumab in this molecular subset was based on the results of a pre-planned retrospective analysis of the Keynote-158 trial in patients with TMB ≥ 10 mutations/Mb, showing ORR of 29% among the 102 patients included in the efficacy analysis. Notably, among the five patients with NEN (primary site not specified), ORR was 40% [116,147]. Table 2 summarized potential agnostic targets for the treatment of UPO-NEN.

## 6. Discussion

In recent decades, the incidence and prevalence of NENs has dramatically risen across all primary sites, stages, and grades, mainly as a result of increased detection rates and advances in systemic therapies. In this scenario, a non-negligible proportion (up to 22% of cases) is represented by sufferers of UPO-NENs, a poor prognostic group with largely unmet clinical needs [7]. UPO NENs present, by definition, with advanced or metastatic disease. No standard therapeutic algorithms are defined, as this population is usually poorly represented in registration randomized phase III trials. Current guidelines suggest that treatment of UPO-NENs should be based on the presumptive site of origin, tumor clinical-pathological characteristics, disease burden, and the patient’s conditions and comorbidities, thus requiring a case-by-case therapeutic individualization [21,22].

The differentiation between well- and poorly differentiated UPO-NEN represents one of the main criteria to orient therapeutic choices. This dichotomous morphological classification reflects underlying differences in terms of genomic characteristics and clinical and biological behavior. Chemotherapy still represents the backbone for the treatment of high-grade, poorly differentiated UPO-NECs, an approach that usually provides deep but short-lasting responses with poor survival outcomes. Attempts to improve survival in this particularly poor prognostic group include treatment intensification with three-drug chemotherapeutic regimens [50,51,52], chemo-immunotherapy combinations [53,63,68], ICIs, or a combination of ICIs and TKIs [63,64,65,66,67,68,69,70,71,73,74,75]. ICI monotherapy has provided unsatisfactory results in unselected patients’ populations in recent clinical trials [63,64,65,66,67]. Although preliminary, interesting activity data have been recently provided about upfront chemoimmunotherapy [53], the use of anti-CTLA-4 plus anti-PD-L1 agents [69,70], and ICI plus TKI combinations [73]. However, such treatment strategies have not been directly compared with standard platinum-based chemotherapy and their development should entail more accurate patient selection based on predictive molecular biomarkers. Indeed, the differences observed in ICI activity between low- and high-grade NENs may rely on the higher TMB and neoantigen burden, and the higher PD-L1 expression in the latter group. To date, conflicting data are available about the role of PD-L1 expression in predicting response to ICIs in UPO-NEN [64,66,109], and the DART-SWOG and CA209–538 trials did not provide activity data of the ipilimumab/nivolumab combination stratified by PD-L1 expression [69,70]. Conversely, MSI-high and TMB-high status in addition to POLε alterations are more reproducible biomarkers of ICI-related benefits across different tumor types [139,146,147].

In recent years, molecular profiling has provided deep insights into the molecular landscape of UPO-NENs, with both diagnostic and therapeutic implications. Overall, about 20% of high-grade NECs may harbor one druggable molecular alteration (including *BRAF* and *KRAS* mutations, *RET*, *ALK*, and *NTRK* 1/2/3 rearrangements, and MSI-high or TMB-high status), so that comprehensive NGS analysis should be advocated in this poor-prognosis subset to orient agnostic target therapies [116].

Although well differentiated UPO-NETs harbor few somatic druggable alterations, molecular profiling platforms providing primary-site specific genomic profiles have been recently developed, with potential clinical applications in the near future [6,120,121]. The integration of molecular biology with standard pathology and imaging approaches may significantly contribute to primary site identification, potentially leading to surgical approaches with radical intent. 

The spectrum of available systemic therapy options for well-differentiated UPO- NETs may range from SSA monotherapy in indolent low-grade NETs, to TKIs, PRRT, or chemotherapy for more aggressive tumors or in the case of a symptomatic disease burden. 

Novel TKIs have shown promising activity and efficacy in the setting of well-differentiated UPO-NETs. Cabozantinib and surufatinib are multitargeted small molecules with high-spectrum biological activity against multiple and non-redundant oncogenic pathways responsible for tumor growth and neoangiogenesis, which yielded improved PFS outcomes compared to placebo in phase III trials including UPO-NETs. In the CABINET and SANET-ep trials, the PFS advantage was evidenced despite poor objective response rates, suggesting that TKI monotherapy may represent a valuable therapeutic option whenever tumor stabilization rather than tumor shrinkage is the therapeutic goal [95,96]. In the case of high tumor burden or symptomatic disease, PRRT or chemotherapy represent the treatments of choice, depending on tumor grade and SSTR expression. Chemotherapy, for example, is a valuable choice for G3 NETs or for NETs with low SSTR on functional imaging [85,86,87,88,89,90,91,97,98,99,100,101,102,103,104,105,106].

Even though immunotherapy showed limited efficacy in well-differentiated NETs, the anti-CTLA-4/anti PD-1 combination or the anti PD-L1 plus TKI combination showed signs of activity in G3 NETs, a subset of tumors with a higher biological aggressiveness and in which multidrug immune modulation may represent a promising therapeutic approach [69,70,73].

**Figure 4:**
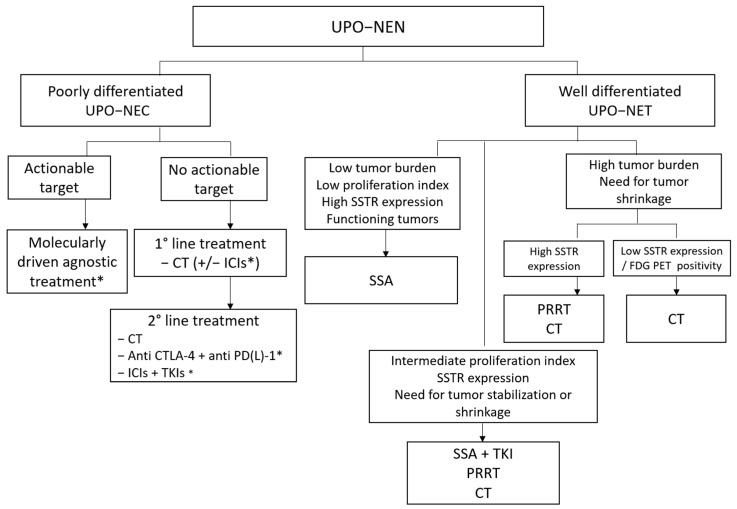
Possible therapeutic algorithm for UPO-NEN. CT: chemotherapy; CTLA-4: anti-Cytotoxic T-Lymphocyte Antigen 4; ICI: immune checkpoint inhibitor; FDG PET: fluorodeoxyglucose positron emission tomography; NEC: neuroendocrine carcinoma; NEN: neuroendocrine neoplasm; NET: neuroendocrine tumor; PD-(L)-1: programmed death (Ligand)-1; PRRT: peptide receptor radioligand therapy; SSA: somatostatin analog; SSTR: somatostatin receptor; TKI: Tyrosine kinase inhibitor; UPO: unknown primary origin. *: under investigation.

Figure 4 depicts a possible future therapeutic algorithm for UPO-NENs, integrating molecular biology profiling, agnostic, and multimodal treatment strategies.

## 7. Conclusions and Future Directions

In conclusion, UPO-NENs represent a rare and heterogeneous disease with limited treatment options. Due to their rarity, we claim the possibility of a change in UPO-NEN management, moving from morphologically driven therapeutic choices, or those presuming the site of origin, to the integration of molecular biology. This opportunity, coupled with agnostic treatments, may pave the way to the definition of personalized therapeutic strategies, particularly when lacking clinical trials specifically drawn for this rare entity. In-development treatment approaches include multidrug combinations (e.g., combinations of ICIs, TKIs, and PRRT) and the implementation of genomic profiling in order to identify druggable molecular alterations, especially in high-grade disease. Improvement in diagnostic and surgical techniques may lead to enhanced locoregional treatments with radical intent. Multidisciplinarity and referral in high-volume centers is of utmost importance to optimize patients’ management.

## Figures and Tables

**Table 1:** Ongoing clinical trials including UPO-NEN.

Trial Identifier	Phase	Treatments	Setting	Primary Endpoint	Status	Estimated Completion Date
NCT04325425 (FOLFIRINEC)	II	mFOLFIRINOX vs. Platinum–Etoposide	1st line GEP or UPO NEC	PFS	Recruiting	September 2024
NCT03980925 (NICE-NEC)	II	Nivolumab/Carboplatin–Etoposide	1st line G3 GEP or UPO NEN	12 months-OS	Active, not recruiting	December 2023
NCT02820857 (BEVANEC)	II	FOLFIRI/Bevacizumab vs. FOLFIRI	Pretreated GEP or UPO NEC	Proportion of pts alive after 6 months	Active, not recruiting	September 2024
NCT03736720	II	Liposomal Irinotecan/Fluorouracil/Leucovorin	Pretreated GEP or UPO NEC	ORR	Active, not recruiting	June 2026
NCT04412629	II	Cabozantinib	Pretreated High Grade NENs including UPO	ORR	Recruiting	November 2024
NCT04525638	II	Nivolumab plus ^177^Lu-Dotatate	Pretreated SSTR positive NET/NEC G3 including UPO	ORR	Recruiting	September 2024
NCT02628067 (Keynote 158)	II	Pembrolizumab	TMB-high /MSI-H solid tumors including NEN	ORR	Recruiting	October 2026
NCT05882058 (DAREON™-5)	II	BI 764532	NECs	ORR, TEAEs	Recruiting	July 2025
NCT02925234 (DRUP)	II	Targeted therapies Basket trial	Solid tumors including NEN	% pts treated based on molecular profile; ORR; G ≥ 3/serious TRAEs	Recruiting	December 2027
NCT04589845 (TAPISTRY)	II	Targeted therapies or immunotherapies Basket trial	Solid tumors including NEN	ORR	Recruiting	September 2032
NCT02568267 (STARTRK-2)	II	Entrectinib	NTRK 1/2/3, ROS1, or ALK rearranged solid tumors including NEN	ORR	Active, not recruiting	April 2025
NCT03157128 (LIBRETTO-001)	I/II	Selpercatinib	RET Fusion-Positive solid tumors including NEN	MTD, RP2D, ORR	Recruiting	February 2026
NCT03037385 (ARROW)	I/II	Pralsetinib	RET altered solid tumors including NEN	MTD, safety, ORR	Active, not recruiting	December 2023
NCT04427787 (LOLA)	II	Lanreotide/ Cabozantinib	Pretreated/not pretreated GEP, thoracic or UPO-NET	Safety, ORR	Recruiting	November 2023
NCT04544098	Early I	intraarterial/ intravenous ^177^Lu-DOTATATE	GEP, Bronchial or UPO NET	nr of pts who completed 2 IA injections; ORR	Recruiting	September 2024
NCT05249114	I	Cabozantinib plus ^177^Lu-Dotatate	SSTR2 positive NET including UPO NET	MTD	Recruiting	December 2027
NCT05554003 (MeTe)	II	Metronomic Temozolomide	NETs including UPO NETs in unfit patients	PFS	Recruiting	December 2024

^177^Lu-DOTATATE: ^177^Lutetium-[DOTA°,Tyr3]octreotate; ALK: Anaplastic lymphoma kinase; G: grade; GEP: gastroenteropancreatic; IA: intraarterial; mFOLFIRINOX: modified folinic acid/5-fluorouracil/irinotecan/oxaliplatin; MSI-H: microsatellite instability high; MTD: maximum tolerated dose; NEC: neuroendocrine carcinoma; NEN: neuroendocrine neoplasm; NET: neuroendocrine tumor; nr: number; NTRK: neurotrophic tyrosine receptor kinase; ORR: overall response rate; OS: overall survival; PFS: progression-free survival; pts: patients; RET: Rearranged during Transfection; RP2D: recommended Phase 2 dose; SSTR: somatostatin receptor; TMB: tumor mutational burden; TEAE: treatment emergent adverse event; TRAE: treatment related adverse event; UPO: unknown primary origin; vs.: versus.

**Table 2:** Novel biomarkers in UPO-NENs and the relative potential therapeutic strategies.

Molecular Target	Targeted Therapies	Level of Evidence	References
BRAF V600E	BRAF-MEK inhibitors	Phase I/II trials Case reports	[122,123,124,125]
KRAS	KRAS G12C inhibitors	Case-reports	[126,127]
ALK	ALK inhibitors	Case-series	[128,129,130,131]
NTRK	NTRK inhibitors, NTRK/ROS1 inhibitors	Phase II trials	[132,133,134,135]
RET	RET kinase inhibitors	Phase I/II trials	[136,137,138]
DLL3	DLL3-targeted antibody-drug conjugate, DLL3-targeting T-cell engager	Phase I/II trials Phase III trials	[140,141,142]
H-MSI	ICIs	Phase II trials	[144,145]
TMB	ICIs	Phase II trials	[147]

ALK: Anaplastic Lymphoma Kinase; DLL3, Delta-like protein 3; H-MSI, high microsatellite instability; KRAS: Kirsten rat sarcoma; ICI: immune checkpoint inhibitor; NTRK, Neurotrophic receptor tyrosine kinase; RET, Rearranged during Transfection; TMB, tumor mutational burden.
